# Antitoxin MqsA Represses Curli Formation Through the Master Biofilm Regulator CsgD

**DOI:** 10.1038/srep03186

**Published:** 2013-11-11

**Authors:** Valerie W. C. Soo, Thomas K. Wood

**Affiliations:** 1 Department of Chemical Engineering; 2 Department of Biochemistry and Molecular Biology, Pennsylvania State University, University Park, Pennsylvania, 16802, USA

## Abstract

MqsA, the antitoxin of the MqsR/MqsA toxin/antitoxin (TA) system, is a global regulator that reduces expression of several stress response genes (e.g., *mqsRA*, *cspD*, and *rpoS*) by binding to the promoter palindromic motif [5′-AACCT (N)_3_ AGGTT-3′]. We identified a similar *mqsRA*-like palindrome [5′-AACCT TA AGGTT-3′] 78 bp upstream of the transcription initiation site in the *csgD* promoter (*p-csgD*). CsgD is a master regulator for biofilm formation *via* its control of curli and cellulose production. We show here that MqsA binds to this palindrome in *p-csgD* to repress *csgD* transcription. As expected, *p-csgD* repression by MqsA resulted in reduced transcription from CsgD-regulated curli genes *csgA* and *csgB* (encoding the major and minor curlin subunits, respectively). Curli production was reduced in colonies and in planktonic cells upon MqsA production. Hence, MqsA directly represses *p-csgD*, and thereby influences curli formation. This demonstrates that TA systems can impact overall cell physiology by fine-tuning cellular stress responses.

Biofilms consist of bacterial populations adherent to each other, and often, to solid/liquid or air/liquid interfaces^1^. In *Escherichia coli* and *Salmonella* spp., a major extracellular component that promotes biofilm formation is curli^2^,^3^,^4^. Curli are thin proteinaceous, amyloid fibers (usually 4–12 nm in width and 100 to 10,000 nm in length)^5^ that were initially identified as a surface organelle in *E. coli* that binds to host fibronectin^6^. Secretion of curli fibers to the bacterial membrane surface requires seven genes in two adjacent divergently transcribed operons: *csgDEFG* and *csgBAC*^7^,^8^. CsgE^9^, CsgF^10^, and CsgG^8^ are accessory membrane proteins for efficient curli secretion, while CsgB and CsgA are structural subunits (curlin) that assemble into mature curli fibers^11^. The role of CsgC in curli biogenesis is less understood, although it has been suggested to participate in redox activity with CsgG^8^. In *E. coli*, both operons are activated by CsgD, a transcriptional regulator belonging to the FixJ/UhpA/LuxR family^7^. In addition to curli expression, CsgD also transcriptionally activates the gene of diguanylate cyclase AdrA, which synthesizes cyclic diguanylate (c-di-GMP)^12^. Both AdrA and c-di-GMP have been implicated in cellulose production^12^,^13^. As both curli and cellulose are components in biofilms, *csgD* regulation is thus an important determinant in microbial adaptation to different environments.

Curli production is highly responsive to environmental fluctuations such as low temperature^6^, low osmolarity^14^, and nutrient limitation^15^. These environmental cues influence the expression of no less than 10 transcriptional factors, which in turn regulate the expression of *csgD*^16^. For instance, *csgD* is activated by RNA polymerase containing the stationary phase sigma subunit σ^S^ (RpoS) during stationary growth phase^17^. This activation is further amplified by a positive feedback loop through CsgD-dependent transcription of *iraP*, which encodes a stabilizing factor for RpoS^18^. Another transcriptional factor, MlrA^19^, also stimulates CsgD expression through a signaling cascade of c-di-GMP generated by YegE and YdaM^20^. At the post-transcriptional level, the 5′ untranslated region of *csgD* mRNA is also a regulatory hotspot^21^. At least four small RNAs [McaS^22^, RprA^23^, and OmrA^24^, OmrB^24^] can directly bind to the 5′ untranslated region of *csgD* to subsequently inhibit its expression. Overall, the multiple regulatory layers for CsgD expression underlie the complex regulation of curli production and biofilm formation.

Bacterial toxin/antitoxin (TA) systems are genetic elements that encode both toxic proteins that disrupt cellular processes, and antitoxins that attenuate this toxicity. TA systems are prevalent, with at least 38 TA systems identified in *E. coli* alone^25^,^26^. Among these, the MqsR/MqsA system is notable for its involvement in persistence^27^, quorum sensing^28^, biofilm formation^28^,^29^, direct control of another TA system^30^, and global regulation through the MqsR toxin^28^ and the MqsA antitoxin^31^. MqsA possesses a C-terminal helix-turn-helix domain^32^ that allows direct binding to a specific palindromic DNA motif [5′-AACCT (N)_3_ AGGTT-3′] found in two copies in the promoter region of *mqsRA*^33^. Upon binding to this palindrome through its C-terminal domain, MqsA controls expression of various genes such as *mqsRA*^33^, *rpoS*^31^, and *cspD*^34^.

Previously, we identified an *mqsRA*-like palindromic motif [5′-AACCT TA AGGTT-3′] in the promoter of *csgD* using a whole-genome bioinformatic search^31^. Here, we show that MqsA binds to this *mqsRA*-like palindrome to repress *csgD* expression, which eventually results in reduced curli formation in *E. coli*. Taken together, MqsA behaves as a negative determinant in biofilm formation and as a regulator of an important regulator (CsgD).

## Results

**Figure 1:**
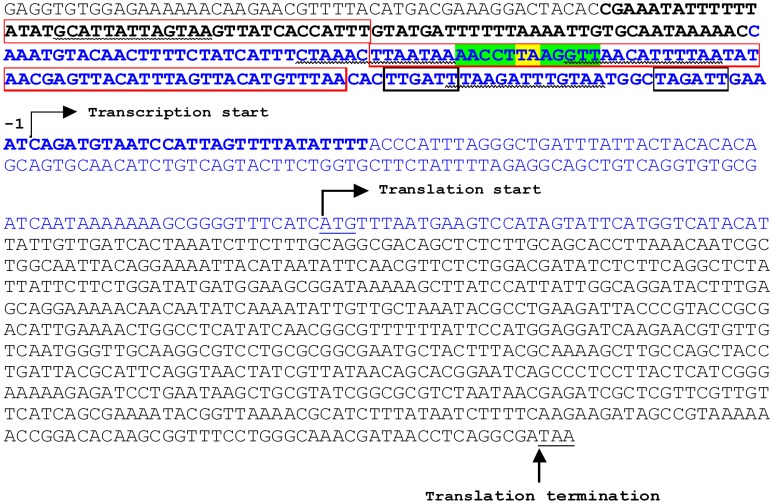
The *csgD* promoter (*p-csgD*) region. The black boxes indicate the −35 and −10 promoter regions. The *mqsRA*-like palindrome that contains the 5′-AACCT (N)_3_ AGGTT-3′ motif is highlighted in green (at position −78 relative to the transcriptional start site) with the spacer in yellow. The sequence in blue was used as the DNA probe (*p-csgD*) for EMSA. Nucleotides in bold indicate the binding site for H-NS, while those boxed in red indicate the binding sites for IHF. Nucleotides with a wavy underline indicate the binding site for CpxR.

We have shown that MqsA is a global regulator that represses *rpoS* transcription by binding at the *mqsRA*-like palindrome of *p-rpoS*^31^. RpoS is the master regulator of stress response^35^. In the presence of oxidative stress, which would normally induce genes positively controlled by RpoS such as those encoding curli, curli/cellulose production was reduced by 13 ± 2 fold in cells producing MqsA, and the *csgD* transcript was decreased by 3 ± 1 fold^31^. Hence, under stressful conditions with overproduction of MqsA, the reduction in curli/cellulose is at least partially a result of a lack of induction of *p-csgD* by RpoS due to MqsA repressing *p-rpoS*. Our previous bioinformatic analysis also identified a *mqsRA*-like palindrome 78 bp upstream of the transcriptional start site of *csgD*^31^ (Fig. 1). We hypothesized that MqsA decreases curli formation by a direct binding to this *mqsRA*-like palindrome to subsequently repress *csgD* at a transcriptional level. Therefore, we examined curli production in the absence of oxidative stress to reduce the effect of RpoS on *p-csgD* which allowed us to see the effect of MqsA directly on *p-csgD*. Note that curli production is RpoS-dependent, so an *rpoS* deletion strain could not be used for phenotypic assays. Since curli is formed in laboratory *E. coli* strains at temperatures between 26°C to 32°C^6^,^36^, 28°C was used here to promote curli formation.

### Curli production is reduced in MqsA-producing strains

**Figure 2:**
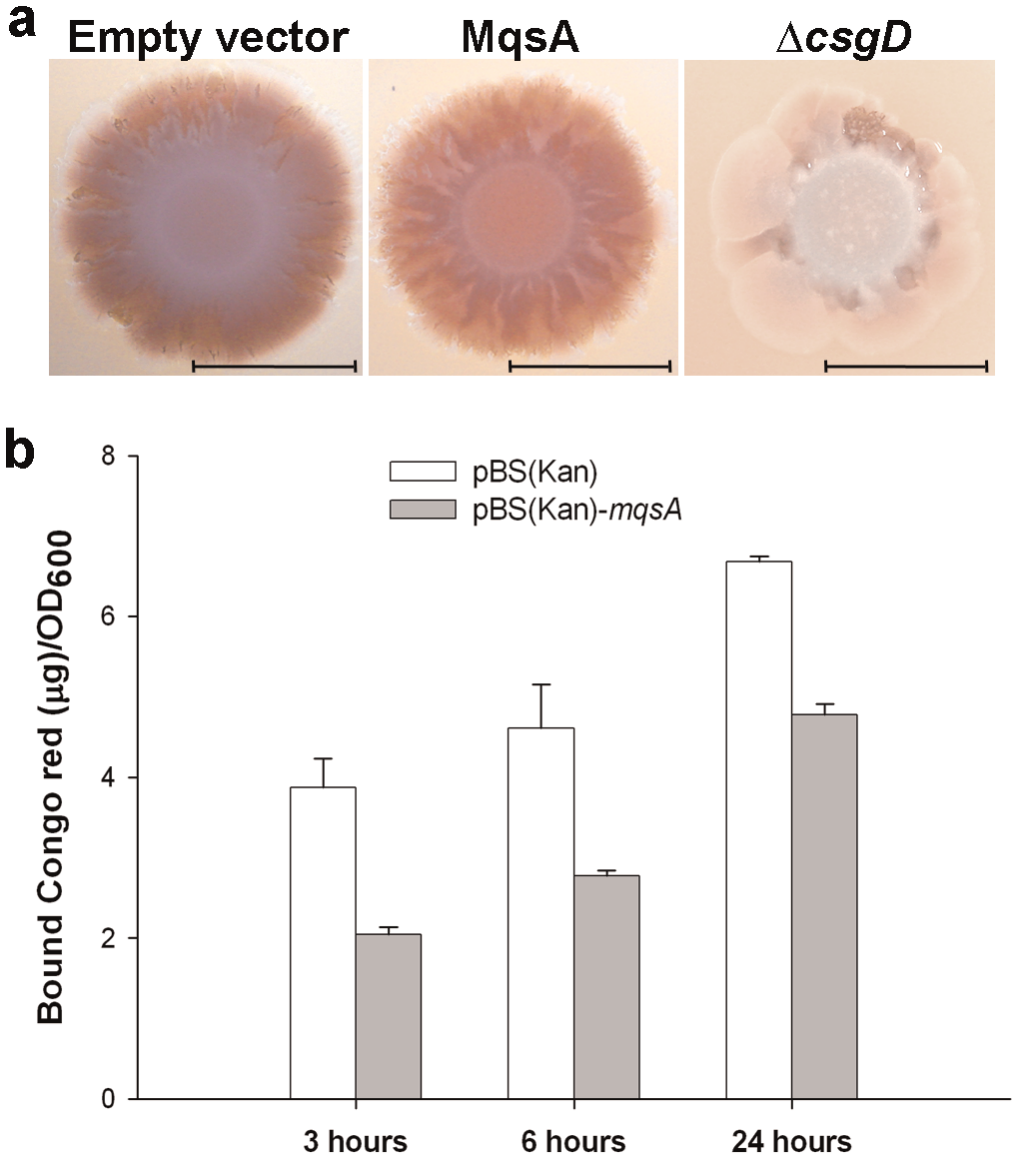
MqsA decreases EPS production. (a) Colony morphology of strains grown on salt-free CR plates containing 1 mM IPTG for 7 days. Red color indicated curli/cellulose production and scale bars represent 1 cm. Empty vector: BW25113 Δ*mqsRA*/pBS(Kan); MqsA: BW25113 Δ*mqsRA*/pBS(Kan)-*mqsA*; and Δ*csgD*: BW25113 Δ*csgD*. (b) The amount of Congo red bound to planktonic cells at various time points. Error bars denote standard deviation (*n* = 2).

As Congo Red (CR) is a dye that binds curli and cellulose^13^, colonies with high amounts of curli will appear red on salt-free agar. Note that *E. coli* K-12 does not produce cellulose^13^,^37^. As expected, the *csgD* deletion strain appeared as white colonies (Fig. 2a). In an *mqsRA* deletion strain, producing MqsA from a plasmid rendered the colonies less red than an isogenic strain harboring the empty plasmid after prolonged incubation (Fig. 2a). By quantifying the amount of CR bound to planktonic cells, we determined that curli production was 1.9 ± 0.2 fold and 1.7 ± 0.2 fold less in the MqsA-producing cells after 3 h and 6 h incubation, respectively (Fig. 2b). Therefore, MqsA reduces CsgD activity *via* two pathways: (i) indirect repression of *p-csgD* through repression of *p-rpoS* under oxidative stress; and (ii) direct repression of *p-csgD* in the absence of oxidative stress.

**Figure 3:**
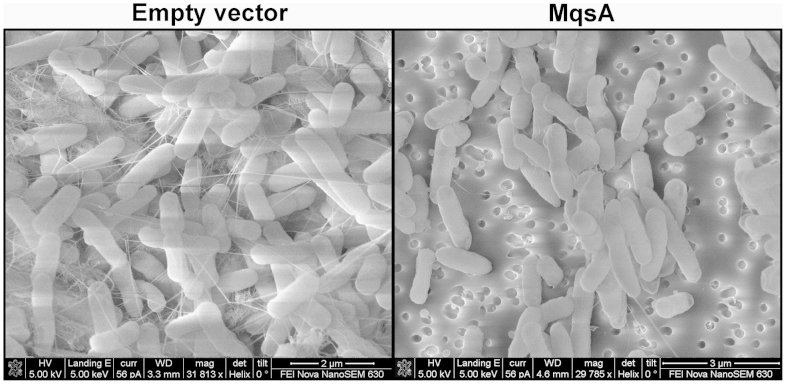
Curli and cellulose are reduced in MqsA-producing cells. Curli production was assayed from cells in 2-day old colonies on agar plates with 1 mM IPTG, and imaged using SEM. Empty vector: BW25113 Δ*mqsRA*/pBS(Kan) and MqsA: BW25113 Δ*mqsRA*/pBS(Kan)-*mqsA*. For each strain, 3 independent colonies were examined, and an image from one representative colony is shown. Scale bars represent 2 μm (left) and 3 μm (right).

To corroborate these results, we further examined the content of curli at cellular level using SEM. In cells harboring the empty plasmid, curli fibers were present (Fig. 3, left panel) in considerable amounts after 2 days of incubation, with curli forming extracellular matrix that traps individual cells to form biofilms. Cells also showed a rougher surface with tiny lumps. In contrast, curli were essentially absent in MqsA-producing cells (Fig. 3, right panel). We estimated that the curli content in 400 MqsA-producing cells was approximately 6 ± 7 fold less in comparison to the same number of cells harboring empty plasmid. Hence, production of MqsA reduces curli production. Given that curli production is positively correlated with biofilm formation, this reduced amount of curli in MqsA-producing strain is consistent with the previous observation that biofilm formation was decreased by 2 fold in cells expressing MqsA^31^.

### Curli-related gene transcripts are reduced in cells expressing MqsA

Since *p-csgD* is repressed by MqsA, we reasoned that genes controlled by CsgD, such as *csgB* (curli-related) and *adrA* (cellulose-related), will also be repressed upon production of MqsA. To investigate this possibility, we tested the expression of *csgB* and *adrA* in various growth conditions using quantitative real-time reverse-transcription PCR (qRT-PCR). *csgB* encodes the minor curlin subunit, while *adrA* (encoding a cyclic diguanylase) is part of the regulatory network in cellulose production. In the five growth conditions tested, the *csgD* transcript in MqsA-expressing cells, whose *mqsRA* loci were deleted, was consistently decreased by 2 to 6 fold, in comparison to cells with the empty plasmid (Table 3). Similarly, *csgB* and *adrA* transcripts were also reduced under the same conditions. The largest reductions in *csgD* and *csgB* transcripts were seen with prolonged MqsA overexpression; in particular, after ~6 h of MqsA overexpression in LB, *csgD* and *csgB* were repressed by nearly 6 fold and 109 fold, respectively. In contrast, *adrA* repression was more apparent when MqsA production was induced for a short duration. Under 30 min induction, *adrA* transcript was decreased by nearly 5 fold, but the reductions were less than 3 fold under long inductions (>1 h). This suggests that CsgD does not activate *csgB* and *adrA* in the same manner^38^, which further implies that curli and cellulose production are regulated differently during biofilm formation.

To demonstrate the direct effect of MqsA on *p-csgD*, we tested the expression of *csgD*, *csgB*, and *adrA* in an *rpoS*-deleted strain. In the absence of RpoS, transcription of *csgD*, *csgB*, and *adrA* in MqsA-overproducing cells remain repressed (Table 3). In comparison with an *rpoS*^+^ strain with MqsA produced from plasmid (BW25113 Δ*mqsRA*/pBS(Kan)-*mqsA*), the *csgD*, *csgB*, and *adrA* transcripts were repressed ~50% less in the *rpoS*-deleted strain with MqsA produced.

To corroborate this direct binding of MqsA to *p-csgD*, we produced MqsA via pCA24N-*mqsA* in MG1655 *Δ*6 R3 P*rpoS* that harbors a mutated *mqsRA*-like palindrome (5′-ACCT TGC TCAC-3′) upstream of chromosomal *rpoS*^31^, and measured the transcription of *csgD*, *csgB*, and *adrA*. In this background, MqsA is unable to affect chromosomal *rpoS* transcription due to the mutated palindrome in the *rpoS* promoter^31^. In comparison to the isogenic strain harboring an empty plasmid, *csgD*, *csgB*, and *adrA* were reduced by ~2 fold upon MqsA production (Table 3). *csgD* transcription was further repressed by nearly 5 fold in MG1655 *Δ*6 R1 P*rpoS*, a strain that harbors the wild-type *mqsRA*-like palindrome (5′-ACCT TGC AGGT-3′) in the *rpoS* promoter^31^. Hence, MqsA represses *csgD* transcription in the absence of its effect on *rpoS* transcription, and there is a greater reduction in transcription of *csgD* when both the promoter of *rpoS* and *csgD* are repressed. Therefore, these results confirm direct *p-csgD* repression by MqsA and demonstrate that repression of curli synthesis by MqsA is a result of repression of both *p-csgD* and *p-rpoS*.

### MqsA binds the *mqsRA*-like palindrome in *p-csgD*

**Figure 4:**
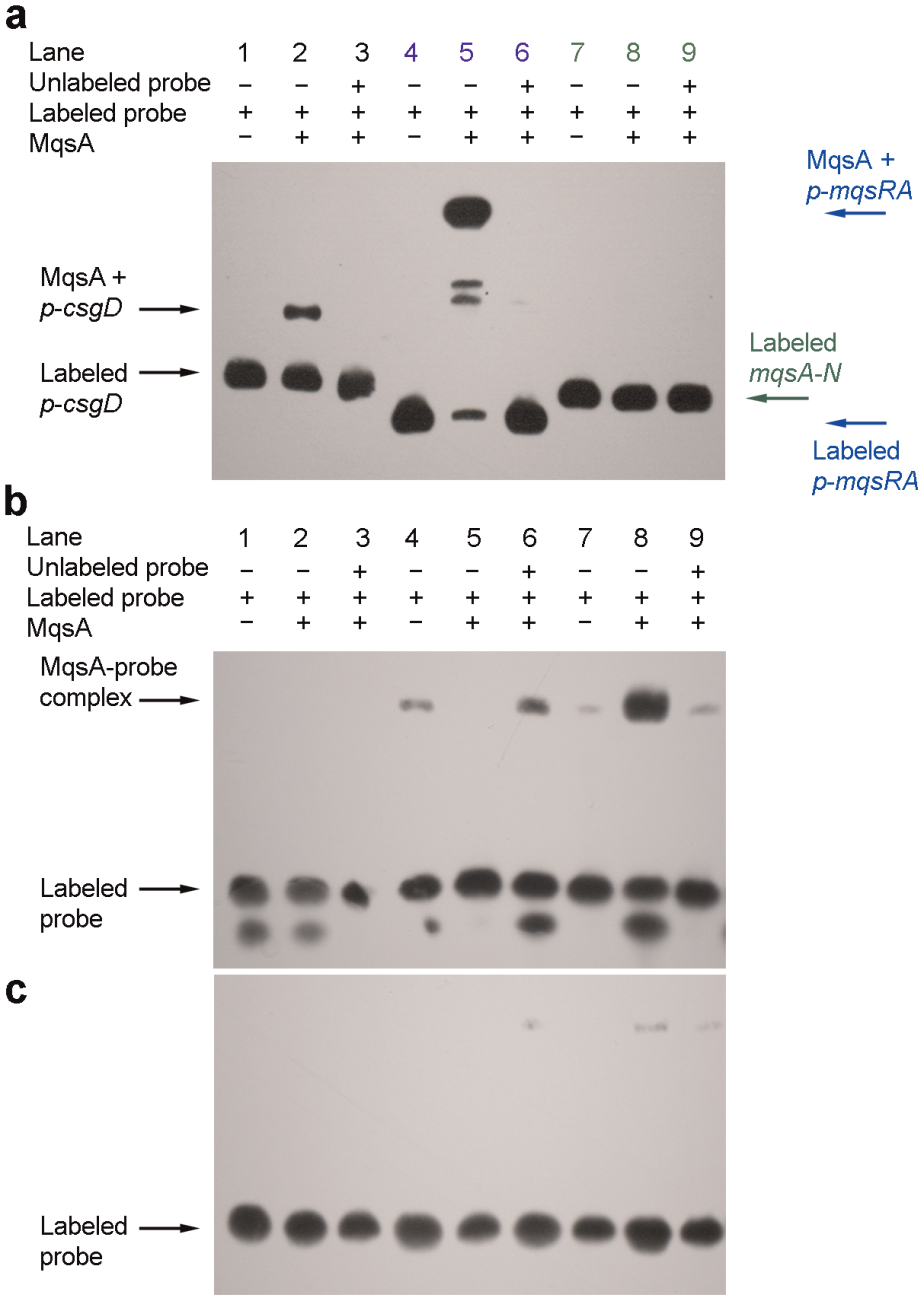
MqsA binds to the *mqsRA*-like palindrome in *p-csgD*. (a) Biotin-labeled *p-csgD* (lanes 1–3, 312 bp), *p-mqsRA* (lanes 4–6, 234 bp, positive control with two palindromes) and *mqsA-N* (lanes 7–9, 287 bp, negative control) were incubated with 200-fold, 100-fold, or 200-fold excess MqsA (lanes 2, 5, 8, respectively). *p-mqsRA* was amplified from the *mqsRA* promoter (which has two palindromes, positive control). *mqsA-N* is the fragment that corresponds to the N-terminus of the coding sequence of *mqsA* (which lacks a palindrome, negative control). (b) Biotin-labeled DNA probes (30-mers containing native *mqsRA*-like palindrome) were incubated with 25-fold, 50-fold, 100-fold, and 200-fold excess MqsA (lanes 2, 4, 6, 8, respectively). Unlabeled probe was added 200 fold in excess (lanes 3, 5, 7, 9). (c) Biotin-labeled DNA probes (30-mers containing mutated *mqsRA*-like palindrome) were incubated with a 25-fold, a 50-fold, a 100-fold, and a 200-fold excess of MqsA (lanes 2, 4, 6, 8, respectively). Unlabeled probe was added 200 fold in excess (lanes 3, 5, 7, 9).

To investigate whether MqsA binds the *mqsRA*-like palindrome in *p-csgD* to mediate gene repression, a 312-bp fragment (*p-csgD*) was amplified from the *csgD* promoter of *E. coli* (Fig. 1), and incubated with MqsA in EMSA reactions. We found that MqsA binds *p-csgD* (Fig. 4a, lane 2), and that this binding could be reversed by adding unlabeled *p-csgD* in excess (Fig. 4a, lane 3). For the positive control, MqsA bound the *p-mqsRA* double palindrome and formed three distinct bands: the most prominent and largest DNA-MqsA band is where MqsA binds both palindromes of *p-mqsRA* whereas the two smaller bands are for single MqsRA binding each of the individual palindromes (Fig. 4a, lane 5).

We investigated the specificity of the MqsA binding to *p-csgD* at the *mqsRA*-like palindrome by incubating MqsA with a 30-bp fragment that corresponds to either the native *mqsRA*-like palindrome (5′-AACCT TA AGGTT-3′) or its mutated counterpart with five nucleotides changed and is not able to form a palindrome (5′-AACCT TA TCACC-3′) (Table 2). MqsA-bound native palindromes in *p-csgD* were shifted upon adding a 50-fold, a 100-fold or a 200-fold excess MqsA (Figure 4b, lanes 4, 6, 8). However, when the *p-csgD* mutated palindrome was used, the binding was drastically reduced (Figure 4c, lanes 4, 6, 8). This shows that MqsA binding to the *mqsRA*-like palindrome in *p-csgD* is specific, and this binding mediates CsgD repression.

## Discussion

Elucidating the synthesis of bacterial curli amyloids, and its regulation, is important for biofilm research, particularly from a clinical perspective. Bacterial curli fibers share structural, biochemical and biophysical properties with protein amyloids^39^, which are notoriously implicated in chronic neurodegenerative disorders such as Alzheimer's disease and Parkinson's disease^40^. Thus, curli formation in bacteria has been proposed as a model system to study amyloid formation and regulation^41^. Similar with eukaryotic amyloids, enterobacterial curli fibers are also able to trigger inflammatory responses in mice^42^. Given that curli amyloids (and other extracellular polymeric substances) are important for the biofilm matrix^43^, curli formation is an attractive target for the development of anti-biofilm and anti-virulence drugs^44^.

Regulation of curli synthesis is complex. Here, we add new insight into the regulation of curli, *i.e.*, MqsA decreases curli formation by repressing *p-csgD*. The 300-bp *p-csgD* has binding sites for over 10 transcription factors^16^ that individually or synergistically respond to various stimuli. Among these, the binding site (the *mqsRA*-like palindrome) for MqsA in *p-csgD* (−78 bp from the transcription start site) overlaps with the binding sites of H-NS (+28 to −201)^45^, CpxR (−68 to −80; −90 to −102)^19^,^45^, and one of the two sites of IHF (−37 to −96)^19^,^45^. In particular, the *mqsRA*-palindrome is flanked by two CpxR binding boxes^45^ (Fig. 1). As both MqsA (this work) and CpxR^46^ negatively regulate curli expression, it is likely that MqsA or CpxR binding will respond to different stimuli (such as oxidative and acid stress for MqsA^31^, or envelope stress for CpxR^47^).

Overall, our results support the notion that TA systems have physiological roles such as biofilm formation^29^,^48^, and antitoxin MqsA behaves as a global regulator intimately related to biofilm formation^31^. When nutrients are plentiful, MqsA increases motility by increasing *flhD* (the master regulator of motility) partly through *rpoS*^31^ inhibition, and partly through *csgD* inhibition; hence, the role of MqsA would be to inhibit biofilm formation in the absence of stress. Under stressful conditions, however, MqsA is degraded by proteases^31^, and MqsR is activated^49^. MqsA degradation leads to derepression of *rpoS* and *csgD*, inhibition of *flhD*, and subsequently, results in increased biofilm^31^ since stress increases biofilm formation^50^. Therefore, this global regulative behavior of MqsA cements the role of antitoxins as far more than regulators of their own loci.

## Methods

### Bacterial strains, plasmids, and culture conditions

All strains and plasmids used in this study are summarized in Table 1. All strains were grown in lysogeny broth (LB, 10 g/L tryptone, 5 g/L yeast extract, and 10 g/L NaCl), unless specifically indicated. Strains MG1655 Δ6 R1 P*rpoS* and MG1655 Δ6 R1 P*rpoS* (Table 1) were cultured using 50 μg/mL kanamycin and 5 μg/mL tetracycline. Kanamycin (50 μg/mL) was also used to maintain pBS(Kan)-based plasmids^51^ and to select for *E. coli* BW25113 Δ*csgD*. Chloramphenicol (30 μg/mL) was used to maintain pCA24N-based plasmids^52^. The C*mqsRA*-f and C*mqsRA*-r primers (Table 2) were used to confirm the *mqsRA* deletion in BW25113 Δ*mqsRA via* PCR and DNA sequencing. Similarly, the *rpoS*-f and *ygbN*-r primers (Table 2) were used to verify the *rpoS* deletion in BW25113 Δ*rpoS via* PCR and DNA sequencing. BW25113 Δ*rpoS* ΔKm^R^ was created by eliminating the Km^R^ cassette in BW25113 Δ*rpoS* Ω Km^R^ 53 using FLP recombinase encoded by pCP20^54^.

### Congo red (CR) assay

Curli production was examined by CR-binding assay at 28°C using agar plates^13^ and planktonic cells^55^. Two μL of each overnight culture was spotted on salt-free CR plates (10 g/L tryptone, 5 g/L yeast extract, 1 mM IPTG, 40 μg/mL CR, 20 μg/mL Coomassie Blue). Plates were incubated for 7 days, and the appearance of red colonies indicates binding to CR. Quantification of CR-binding was performed by measuring the amount of CR binding in planktonic cells. Briefly, 1 mL of each overnight culture was harvested at 13,000 × *g* centrifugation for 2 min, and washed with 1 mL of T-broth (10 g/L tryptone). Cells were resuspended in T-broth containing 1 mM IPTG and 40 μg/mL CR, and were incubated at 28°C for 3 to 24 h. Prior to incubation, an aliquot of cell suspension was removed, and CR was measured spectrophometrically at 490 nm (Abs_490 initial_). At specified time points, cells were harvested at 13,000 × *g* centrifugation for 10 min, and the supernatant was spectrophotometrically measured at 490 nm (Abs_490 unbound_). The Abs_490 bound_ was therefore calculated from Abs_490 initial_ – Abs_490 unbound_, and the amount of CR bound to cells (μg) was obtained from a standard curve constructed using 0 μg to 40 μg of CR dissolved in T-broth. All CR values were normalized using cell densities (OD_600_).

### Scanning electron microscopy (SEM)

Curli formation in cells in colony biofilms^56^ was determined using two-day old colonies grown on salt-free LB agar with 1 mM IPTG at 28°C. Colony cells were suspended gently in 1 mL of 100 mM sodium cacodylate buffer (pH 7.4), and collected by filtering^57^ with a 0.22-μm polycarbonate filter (GE Healthcare, Little Chalfont, UK). Cell-containing filters were placed in specimen baskets. Filters were fixed with 2.5% (v/v) glutaraldehyde and 2% (v/v) formaldehyde in sodium cacodylate buffer for 15 min, followed by three washes with buffer alone for 5 min each wash. Filters were then fixed with 1% (w/v) osmium tetroxide in sodium cacodylate buffer for 30 min in dark. After two washes with buffer alone, filters were dehydrated in a graded ethanol series (50%, 70%, 85%, 95% and 3 times with 100% ethanol, for 5 min each). The dehydrated filters were dried using a critical point dryer (Bal-tec CPD-030) with liquid carbon dioxide as a transitional fluid. After affixing the filters on SEM stubs using double-sided carbon adhesive tape, the filters were sputter coated with white gold (Au/Pd) for 3 min (Bal-tec SCD-050 Sputter Coater). Coated specimens were examined with a field emission SEM (FEI NanoSEM 630 FESEM) operating under low vacuum mode (5 kV).

### RNA isolation and qRT-PCR

Total RNA was isolated from planktonic cells using a Qiagen RNeasy Mini kit, as described previously^58^. IPTG (1 mM) was used to produce MqsA in planktonic cells (IPTG was also added to the empty plasmid controls) for various durations and in various growth medium at 28°C (Table 3). RNA integrity was checked by agarose gel electrophoresis, and by the Abs_260_/Abs_280_ ratio of 1.85 to 2.25. qRT-PCR was performed according to manufacturer's instructions (*Power* SYBR Green RNA-to-C_T_ *1-Step* kit, Life Technologies, Carlsbad, CA) using 100 ng of total RNA as the template. Primers were annealed at 60°C, and *rrsG* was used to normalize all data. The specificity of all qRT-PCR primers (Table 2) was verified using normal PCR. Fold changes in various gene transcripts in MqsA-producing strain in relative to strain harboring empty vector were calculated using the 2^−ΔΔCt^ formula^59^.

### Electrophoretic mobility shift assay (EMSA)

Gene promoters were PCR-amplified from the genomic DNA of *E. coli* BW25113 and 3′-labeled with biotin (Biotin 3′-End DNA Labeling kit, Thermo Scientific, Waltham, MA). Primers *p*-*mqsR*-f and *p-mqsR*-r (Table 2) were used to amplify *p-mqsRA* (corresponds to position 3166541 bp to 3166774 bp of *E. coli* MG1655; Genbank accession number: U00096.2). Primers EMSA-*csgD*-f and EMSA-*csgD*-r (Table 2) were used to amplify *p-csgD* (corresponds to position 1102383 bp to 1102694 bp of *E. coli* MG1655). The *mqsA-N* fragment (that corresponds to the 253 bp at the 5′ coding sequence of *mqsA*) was generated by primers *mqsAep*-f and *mqsA-N*-r (Table 2). To investigate MqsA binding specificity, complimentary oligonucleotides with their 3′ end labeled with biotin were synthesized, solubilized, and annealed as previously described^31^. Palindrome 1 of *p-mqsRA* is at position 3166595 bp to 3166609 bp; palindrome 2 is at position 3166629 bp to 3166639 bp; Genbank accession number: U00096.2).

Binding reactions were performed in 10 mM Tris-HCl (pH 7.5), 50 mM KCl, 1 mM DTT and 1 μg of poly (dI.dC). Briefly, 30 to 50 fmol of labeled DNA probe was incubated with purified MqsA in excess at ambient temperature for 1 h. Purified MqsA was sequentially obtained by His_6_-tagged purification, removal of His_6_ tag using TEV cleavage, and size exclusion chromatography^32^. The reactions were electrophoresed on a 6% DNA retardation gel (Life Technologies, Carlsbad, CA) at 100 V in 0.5× TBE buffer for 90 min. Samples in the gel were electroblotted onto a nylon membrane (GE Healthcare, Little Chalfont, UK) at 380 mA in 1× TBE buffer for 1 h. The membrane was UV-crosslinked at 302 nm for 20 min, and detection was carried out using the protocol described in the Chemiluminescent Nucleic Acid Detection Module (Thermo Scientific, Waltham, MA).

## Figures and Tables

**Table 1:** Bacterial strains and plasmids used in this study

Strains or plasmids	Description	Source
***E. coli* K-12**		
BW25113	*lacI*^q^ *rrnB*_T14_ Δ*lacZ*_WJ16_ *hsdR514* Δ*araBAD*_AH33_ Δ*rhaBAD*_LD78_	53
BW25113 Δ*mqsRA*	BW25113 Δ*mqsRA* ΔKm^R^	34
BW25113 Δ*csgD*	BW25113 Δ*csgD* Ω Km^R^	53
BW25113 Δ*rpoS* ΔKm^R^	BW25113 Δ*rpoS* ΔKm^R^	This study
MG1655 Δ6 R1 P*rpoS*	MG1655 Δ*mazEF* Δ*relBEF* Δ*chpB* Δ*yefM-yoeB* Δ*dinJ-yafQ* Δ*mqsRA* Δ*lacZYA* Ω Km^R^ P*_rpoS_*::*lacZ*-Tet^R^ P*_rpoS_*::*rpoS*	31
MG1655 Δ6 R3 P*rpoS*	MG1655 Δ*mazEF* Δ*relBEF* Δ*chpB* Δ*yefM-yoeB* Δ*dinJ-yafQ* Δ*mqsRA* Δ*lacZYA* Ω Km^R^ P*_rpoS_*::*lacZ*-Tet^R^ P*_rpoS-M_*::*rpoS*	31
**Plasmids**		
pBS(Kan)	Km^R^	51
pBS(Kan)-*mqsA*	Km^R^; P*_lac_*::*mqsA*^+^	34
pCA24N	Cm^R^; *lacI*^q^	52
pCA24N-*mqsA*	Cm^R^; *lacI*^q^, P*_T5-lac_*::*mqsA*^+^	52
pCP20	Ap^R^, Cm^R^; FLP^+^, λ *c*I857^+^, λ *p_R_* Rep^ts^	54

Km^R^, Ap^R^, Cm^R^, and Tet^R^ denotes kanamycin, ampicillin, chloramphenicol and tetracycline resistance, respectively.

**Table 2:**
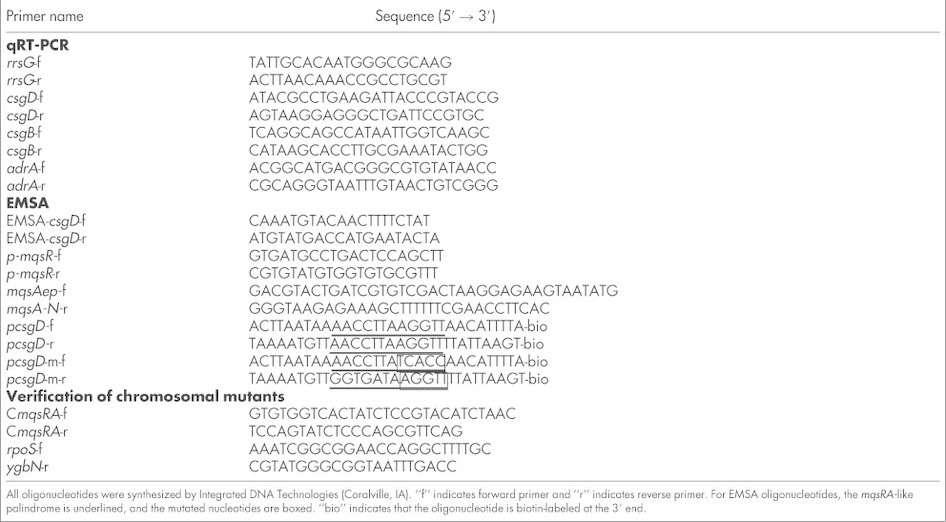
Oligonucleotides used for qRT-PCR, EMSA and for verification of chromosomal mutants

**Table 3:** Summary of qRT-PCR results

Condition				Fold change		
Host	OD_600_ at induction	Growth medium	Induction duration	*csgD*	*csgB*	*adrA*
Plasmids: pBS(Kan)-*mqsA* vs. pBS(Kan)						
BW25113 Δ*mqsRA*	1.0	LB	1.0 h	−3.4 ± 1.2	−3.0 ± 1.3	−2.6 ± 1.2
BW25113 Δ*rpoS* ΔKm^R^	1.0	LB	1.0 h	−1.5 ± 1.2	−1.4 ± 1.3	−1.4 ± 1.2
BW25113 Δ*mqsRA*	1.0	LB	5.5 h	−5.7 ± 1.4	−109.2 ± 1.4	−1.1 ± 1.5
BW25113 Δ*mqsRA*	1.0	Salt-free LB	5.5 h	−1.6 ± 1.4	−3.6 ± 1.4	−1.2 ± 1.4
BW25113 Δ*mqsRA*	6.0	Salt-free LB	0.5 h	−2.9 ± 1.4	−3.2 ± 1.4	−4.6 ± 1.4
BW25113 Δ*mqsRA*	0.3	M9/glucose + 2.5% LB^12^	1.0 h	−3.2 ± 1.1	−3.0 ± 1.2	−2.4 ± 1.2
Plasmids: pCA24N-*mqsA* vs. pCA24N						
MG1655 Δ6 R1 P*rpoS*	0.5	LB	3.0 h	−4.7 ± 1.1	−1.7 ± 1.2	−1.7 ± 1.3
MG1655 Δ6 R3 P*rpoS*	0.5	LB	3.0 h	−2.1 ± 1.3	−2.0 ± 1.2	−1.9 ± 1.2

Means and standard deviations for duplicate reactions are indicated. Negative fold changes denote gene repression for cells overproducing MqsA vs. the empty vector. IPTG (1 mM) was added to the empty plasmids (pBS(Kan) and pCA24N) and used to induce expression of MqsA from pBS(Kan)-*mqsA* and pCA24N-*mqsA.*
